# Crude Extract Versus Tablet Formulation of *Artemisia herba alba*: Dual Strategy for Effective and Quality-Preserving Control of the Rice Weevil, *Sitophilus oryzae*

**DOI:** 10.3390/insects17060574

**Published:** 2026-05-31

**Authors:** Hanaa Elbrense, Marwa N. El-Nahass, Karim Samy El-Said, Mohamed. T. Yassin

**Affiliations:** 1 Department of Zoology, Faculty of Science, Tanta University, Tanta 31527, Egypt; mohamed.taha@science.tanta.edu.eg; 2 Department of Chemistry, Faculty of Science, Tanta University, Tanta 31527, Egypt; marwa.elnahas@science.tanta.edu.eg (M.N.E.-N.); kareem.ali@science.tanta.edu.eg (K.S.E.-S.)

**Keywords:** botanical insecticides, phytochemical-based pest control, *Artemisia herba-alba*, *Sitophilus oryzae*, stored-grain protection, formulation strategy

## Abstract

Botanical insecticides represent a promising alternative to synthetic chemicals for controlling stored-grain pests. However, their use is limited by formulation-related challenges that affect grain quality. This study conducted a comparative evaluation of the insecticidal effectiveness of *Artemisia herba-alba* crude extract and a solidified matrix formulation (Tablet-like formulation), against *Sitophilus oryzae*. The formulation involved embedding the extract in a cellulose–starch base. The evaluation included insect mortality, repellency behavior, oxygen consumption, and selected biochemical parameters. Chemical profiles were analyzed using gas chromatography–mass spectrometry (GC–MS). The crude extract demonstrated strong insecticidal activity, achieving 100% mortality at 10,000 ppm after 72 h. In contrast, the tablet-like formulation showed reduced mortality (33%) under the same conditions. Similar trends were observed in repellency tests, with the crude extract showing higher immediate activity (100%) compared to the tablet formulation (67.44%). Both treatments significantly decreased oxygen consumption and levels of lipase, α-amylase, and neuropeptide F. Only the crude extract affected protease and monoamine oxidase activities. GC–MS analysis revealed that, despite variations in the number and relative abundances of compounds detected, both formulations shared several major constituents. In sum, the crude extract showed superior performance in several parameters, particularly in rapid activity, while both forms exhibited comparable effects in other responses. These findings indicate that the intended application should guide formulation selection, with the crude extract is more suitable for rapid pest control, while the tablet-like formulation may be better for long-term protection and preservation of grain quality during storage.

## Simple Summary

This study highlights that the effectiveness of botanical insecticides cannot be generalized based solely on formulation type. Despite the reported advantages of controlled-release tablet formulations, the crude extract of *Artemisia herba-alba* exhibited superior efficacy against *Sitophilus oryzae*, particularly in terms of insect mortality, while the tablet-like formulation showed comparable effects in repellency and certain physiological responses. These findings emphasize that formulation selection should be guided by the intended pest management objective, with crude extracts being more suitable for rapid control and tablet formulations offering potential benefits for preventive protection and grain preservation during storage.

## 1. Introduction

Stored grains are among the most vital strategic commodities for global food security, especially in developing countries where cereal-based diets sustain large populations [1]. The Food and Agriculture Organization (FAO) predicts that stored-product pests could contribute up to 70% of global food losses by 2050 [2]. Infestations not only reduce the quantity but also the nutritional and commercial quality of grains [3]. Rice (*Oryza sativa* L.) (Poales: Poaceae), the world’s second most important cereal crop after maize, accounts for an estimated annual global production of about 510 million tons of milled rice. Therefore, protecting stored rice remains a key priority for maintaining food reserves [4].

The rice weevil, *Sitophilus oryzae* (L.) (Coleoptera: Curculionidae), is one of the most destructive pests of stored rice that feeds on whole cereal grains [5]. Females bore into grains, lay a single egg, and seal the cavity with secretions, while larvae consume the kernel completely before pupation [6]. Under moderate storage conditions, infestations can cause 10–65% grain losses, which may exceed 80% in poorly managed or long-term storage facilities [7].

Although synthetic insecticides are widely used for *S. oryzae* control, their indiscriminate application raises concerns regarding human health, environmental pollution, pest resistance, and chemical residues [8]. These issues have increased interest in botanical insecticides, which are biodegradable, eco-friendly, and safer for humans and non-targets [9,10,11,12].

The desert wormwood, *Artemisia herba-alba* Asso (Asterales: Asteraceae), is a perennial shrub widely distributed across Western Asia, Southwestern Europe, and parts of the Middle East, including Iran and Egypt [13]. It exhibits multiple biomedical activities, including antidiabetic, antibacterial, antiviral, antioxidant, and antispasmodic effects [14,15]. Recent studies have highlighted its potential as an insecticide, repellent, and growth inhibitor against pests such as red flour beetle, *Tribolium castaneum* (Hbst.) (Coleoptera: Tenebrionidae)*;* Egyptian cotton leafworm, *Spodoptera littoralis* (Boisduval) (Lepidoptera: Noctuidae); codling moth, *Cydia pomonelle* (L.) (Lepidoptera: Tortricidae); and the common house mosquito, *Culex pipiens* L. (Diptera: Culicidae) [16,17,18,19]. Despite these promising bioactivities, the practical application of *A. herba-alba* essential oils and crude extracts for stored-grain protection remains limited. These limitations are mainly attributed to strong odor and taste, surface oiliness, and most importantly, the rapid degradation and volatilization of active compounds, which reduce residual efficacy and necessitate repeated applications [20,21]. Rapid release of bioactive constituents has also been reported as a key factor limiting the field applicability of botanical insecticides [22].

In response to these limitations, formulation-based strategies have been increasingly explored [22]. Such approaches have been suggested to improve the botanical efficacy and potentially reduce negative impacts on grain sensory quality. Chowdhury et al. [23] reported that different formulations may modify the stability and availability of bioactive compounds. Therefore, comparing different formulation forms of the same botanical extract is essential for identifying the most suitable delivery system for practical implementation and effective pest management.

Tablet formulations have been widely recognized as a promising delivery system due to their improved operational convenience, enhanced storage stability, and accurate dose standardization, which collectively support their potential applicability [24,25]. Several studies have reported encouraging outcomes using tablet-based botanical formulations. For example, Kathirvelu et al. [26] demonstrated the effectiveness of tablet formulations derived from sweet basil, *Ocimum basilicum* L. (Lamiales: Lamiaceae), against *S. oryzae*. Additionally, Iqbal et al. [27] investigated biowaste-derived green insecticide formulations and reported their effectiveness against the German cockroach, *Blattella germanica* (L.) (Blattodea: Ectobiidae). More broadly, advances in formulation technologies, including nanoformulations and soluble tablet systems, have shown promise in enhancing the delivery and performance of botanical insecticides [28,29].

However, whether such formulation approaches consistently enhance biological performance relative to the corresponding crude extracts remains insufficiently understood. In particular, limited information is available regarding how converting *A. herba-alba* crude extract into tablet formulation influences the biological and physiological responses of *S. oryzae*, as well as their potential effects on the relative abundance and composition of key chemical constituents. Accordingly, the present study aims to comparatively evaluate the insecticidal efficacy of crude extract and the solidified matrix formulation (tablet-like form) of *A. herba-alba* against *S. oryzae*. The assessment extends beyond mortality to include repellency responses, respiratory metabolic activity, and the modulation of key digestive enzymes, as well as neurophysiological targets including monoamine oxidase (MAO) and neuropeptide F(NPF). Furthermore, gas chromatography–mass spectrometry (GC–MS) analysis was conducted to characterize and compare the phytochemical profiles of both preparations, thereby providing a more comprehensive interpretation of their observed bioactivity.

## 2. Materials and Methods

### 2.1. Plant Material and Extraction

*Artemisia herba-alba* was collected from Siwa Oasis, Egypt. The plant material was identified and authenticated by a taxonomist at the Department of Botany, Faculty of Science, Tanta University, Egypt, and a voucher specimen was deposited in the Tanta University Herbarium (TAN-147). The aerial parts were processed according to Nortjie et al. [30]. Briefly, the plant material was washed with distilled water and air-dried at room temperature for 20 days until completely dry, as determined by visual assessment. Then pulverized using a mechanical grinder (Fisher Scientific, Waltham, MA, USA). Approximately 200 g of powdered plant material was macerated in 2000 mL of 70% (*v*/*v*) ethanol (1:10 *w*/*v*) at room temperature (28–33 °C) for 72 h under continuous shaking using an orbital shaker (SHK-00710, Infitek Co., Ltd., Shanghai, China). The extract was filtered, and the solvent was removed under reduced pressure at 40 °C using a rotary evaporator (Heidolph Instruments, Schwabach, Germany), yielding a concentrated crude extract.

### 2.2. Tablet Formulation

The crude plant extract was completely dried before formulation. Microcrystalline cellulose (MCC) (Sigma-Aldrich, St. Louis, MO, USA) was used as a binder, while starch (Al-Monairy Corn Products, 10th of Ramadan City, Egypt) functioned as a disintegrant. Laboratory-scale solid formulations were prepared following Kathirvelu et al. [26] with minor modifications. A constant excipient ratio of 55:40:5 (extract:MCC:starch) was maintained within each formulation group. Five extract-equivalent amounts per unit (0.1, 1, 10, 100, and 1000 mg) were independently prepared to evaluate concentration-dependent insecticidal activity against *S. oryzae*. For higher extract-equivalent amounts (≥10 mg per unit), the components were manually blended using geometric dilution to ensure uniform distribution of the extract within the excipient matrix. For low extract-equivalent amounts (0.1 and 1 mg per tablet), a batch-based approach was employed to ensure content uniformity. Distilled water (q.s.) was added to obtain a cohesive, moist blend suitable for manual molding. The cohesive blend was manually shaped into solid units using consistent handling procedures. The term “tablet-like formulation” is used descriptively to refer to molded solid units and does not imply a pharmaceutically compressed dosage form. Drying was performed at room temperature (28–30 °C) for 72–96 h until visually dry and structurally stable. Blank formulations without plant extract were used as negative controls.

The physicochemical properties of the prepared *A. herba-alba* tablet-like formulations were evaluated to characterize their physical integrity as laboratory-scale plant-based delivery systems [26,27], not as pharmaceutically standardized tablets. Mechanical stability was assessed through hardness measurement, representing the resistance of the molded units to applied compressive force. Ten units (*n* = 10) from each formulation were randomly selected and individually tested using a digital hardness tester (Erweka GmbH, Langen, Germany) until structural failure. Resistance to mechanical stress was further evaluated using a friability test, where ten pre-weighed units were subjected to controlled rotational stress (100 revolutions at 25 rpm) in a friabilator (PTF 100, Pharma Test Apparatebau AG, Hainburg, Germany). Samples were then dedusted and reweighed, and weight loss was recorded as an indicator of structural integrity. Moisture-related behavior was assessed as a physical stability indicator based on weight variation under desiccation conditions [31,32]. Briefly, ten pre-weighed units were exposed to silica gel in a desiccator at 30–32 °C for 4 h, and subsequent weight change was recorded as an indirect measure of residual water loss [33].

### 2.3. Insect Host and Culture

Adults of *S. oryzae* were obtained from laboratory colonies maintained at the Sakha Agricultural Research Station, Kafr El-Sheikh Governorate, Egypt. The insects were continuously reared on rice grains under controlled conditions (28–30 °C, 65–70% relative humidity, and 12L:12D photoperiod). These conditions were maintained throughout all the subsequent bioassays. Cohorts of uniformly aged adults (5–7 days old) were selected for the bioassay experiments to ensure consistency across treatments. All procedures related to the rearing and handling of *S. oryzae* were conducted in accordance with ethical standards approved by the Research Ethics Committee, Faculty of Science, Tanta University, Egypt, under the Institutional Animal Care and Use Committee (IACUC) approval (protocol no. SCI-TU-0526).

### 2.4. Bioassays

#### 2.4.1. Toxicity Assay for Crude Extract

The toxicity of *A. herba-alba* crude extract was evaluated using a surface exposure assay following Adak and Mukherjee [34] and Rajarushi et al. [35], with slight modifications. The crude extract was prepared in distilled water to obtain five working concentrations (10,000, 1000, 100, 10, and 1 ppm). For each treatment, Whatman No. 1 filter paper (12 cm diameter) was placed in a Petri dish of the same size, and 1 mL of the respective solution was evenly applied onto the surface. Treated filter papers were allowed to air-dry for 2 min to permit partial evaporation of water and uniform deposition of the crude extract on the surface, while minimizing potential loss of volatile constituents. Twenty mixed-sex adults of *S. oryzae* were introduced onto each treated filter paper. Petri dishes were covered with ventilated lids to prevent insect escape. Control insects were introduced to filter papers treated with distilled water only. Each treatment was replicated five times and conducted under the same environmental conditions as described in Section 2.3. Mortality was recorded after 24, 48, and 72 h of exposure. Insects were considered dead when no movement was observed after gentle probing with a fine brush, indicating a complete lack of locomotor or reflex responses. Median lethal concentrations (LC_50_) were estimated using Finney’s probit analysis spreadsheet calculator (Excel-based tool, 2021) [36]. A sub-lethal concentration corresponding to 10% of the 24 h LC_50_ was calculated and used in all subsequent bioassays, following the method described by Al Naggar et al. [37].

#### 2.4.2. Toxicity Assay for Tablet-like Formulation

The toxicity of the tablet-like formulations was evaluated using a surface exposure assay adapted from Kathirvelu et al. [26]. Five extract-equivalent amounts (0.1, 1, 10, 100, and 1000 mg) of *A. herba-alba* were tested to ensure direct comparability with the crude extract assay. For each treatment, twenty mixed-sex adults of *S. oryzae* were placed on a Whatman No. 1 filter paper (12 cm diameter) inside a Petri dish of the same size, and the corresponding tablet-like formulation was then placed in direct contact with the insects on the paper surface. Petri dishes were covered with lids provided with small perforations to allow limited air exchange while minimizing uncontrolled loss of volatile constituents. Control groups included blank tablets and untreated insects. Each treatment was replicated five times. Mortality was recorded at 24, 48, and 72 h post-exposure. Median lethal concentrations (LC_50_) were estimated using Finney’s probit analysis spreadsheet calculator (Excel-based tool, 2021) [36]. A sublethal concentration corresponding to 10% of the 24 h LC_50_ value was calculated and subsequently used in all bioassays, following the method described by Al Naggar et al. [37].

#### 2.4.3. Repellency Test

Repellency bioassays were conducted following Bouzeraa et al. [38]. A Whatman No. 1 was divided with a pencil line and secured at the bottom of a 12 cm Petri dish using a small drop of adhesive. One half was treated with 1 mL of *A. herba-alba* crude extract at a sublethal concentration, while the other half received 1 mL of distilled water as a control. The treated paper was allowed to air-dry for 2 min before releasing twenty adult *S. oryzae* (mixed sex) along the central dividing line. Petri dishes were covered with non-perforated lids coated internally with a thin layer of Vaseline to prevent insect escape and interference with the assay. For the tablet assay, one half of the filter paper received a tablet-like formulation containing the sublethal concentration of the extract, while the opposite half received a blank tablet. Each treatment was replicated three times, and insect distribution on each half was recorded at 1, 2, 3, 4, 5, and 24 h. The percent repellency (PR) was calculated according to Talukder and Howse [39] as follows:

PR (%) = [(Nu − Nt)/(Nu + Nt)] × 100

where Nu and Nt represent the number of insects on untreated and treated halves, respectively.

#### 2.4.4. Oxygen Consumption Rate Assay

The oxygen consumption rate of *S. oryzae* adults exposed to *A. herba-alba* extract and tablet-like formulation was measured using a modified closed respirometry system [40]. The setup consisted of a glass test tube (5 cm in length) sealed with a rubber stopper containing a calibrated capillary tube (1.15 mm internal diameter, 3 cm length). A droplet of diluted ink was placed at the open end of the capillary to act as a visual indicator of gas displacement. Five mixed-sex adults, previously exposed for 24 h to the crude extract or tablet-like formulation at the sublethal concentration, were introduced into the tube. A small piece of filter paper impregnated with 0.1 mL of 10% potassium hydroxide (KOH) was included to absorb CO_2_ produced during respiration, allowing the observed volume change to be primarily attributed to oxygen consumption. After 60 min, the displacement of the ink droplet along the capillary tube was recorded. Each treatment, including the control, was replicated five times. Oxygen consumption was calculated using the formula: V = πr2h, where V: Volume of gas consumed (in µL), π: a constant approximately equal to 3.1416, r: Inner radius of the capillary tube (in mm), h: Distance traveled by the ink marker inside the capillary (in mm). The resulting values were divided by the number of insects (*n* = 5) to express oxygen consumption on a per insect basis.

### 2.5. Biochemical Measurements Assay

#### 2.5.1. Sample Preparation

Insects were pre-exposed for 24 h to either *A. herba-alba* tablet-like formulation or crude extract at the sublethal level. Following treatment, 50 live insects were collected from each control and treated group for enzymatic analyses. Samples were homogenized on ice in cold phosphate-buffered saline (PBS) at a 1:10 (*w*/*v*) ratio using a glass–Teflon homogenizer (Fisher brand, Waltham, MA, USA). The homogenates were centrifuged at 12,000× *g* for 15 min at 4 °C, and the resulting supernatants were carefully collected and kept on ice until further analysis. Each treatment was replicated five times. Total protein content was determined using the Bradford method [41].

#### 2.5.2. Lipase Activity

This assay was conducted according to Winkler and Stuckmann [42]. The substrate p-nitrophenyl palmitate (p-NPP) (Sigma-Aldrich, St. Louis, MO, USA) was first dissolved in isopropanol and then emulsified in 50 mM Tris–HCl buffer (pH 8.0) containing 0.5% Triton X-100 (Sigma-Aldrich, St. Louis, MO, USA). The enzymatic reaction was initiated by adding 20 µL of insect homogenate to 180 µL of substrate emulsion in each microplate well. The reaction mixture was incubated at 37 °C for 30 min, and absorbance was measured at 405 nm. Lipase activity was expressed as the amount of enzyme required to release 1 µmol of p-nitrophenol per minute under the assay conditions and was normalized to protein content (U mg^−1^ protein).

#### 2.5.3. α-Amylase Activity

The reaction mixture was prepared by mixing 20 µL of insect homogenate with 180 µL of 1% soluble starch dissolved in 50 mM sodium phosphate buffer (pH 6.9), followed by incubation at 37 °C for 10 min [43]. The reaction was terminated by adding 200 µL of DNS (3,5-dinitrosalicylic acid) reagent (El-Nasr Pharmaceutical Chemicals Co., Abu Zaabal, Qalyubia, Egypt). The mixture was then boiled for 5 min and subsequently cooled to room temperature. Absorbance was measured at 540 nm, and enzyme activity was determined using a maltose standard curve. Results were expressed as µmol of maltose released per minute per milligram of protein.

#### 2.5.4. Protease Activity

A reaction mixture containing 20 µL of insect homogenate and 180 µL of 1% azocasein (Sigma-Aldrich, St. Louis, MO, USA) prepared in 50 mM Tris–HCl buffer (pH 8.0) was incubated at 37 °C for 30 min [44]. The reaction was terminated by adding 200 µL of 10% trichloroacetic acid (TCA) (El-Nasr Pharmaceutical Chemicals Co., Abu Zaabal, Qalyubia, Egypt), followed by centrifugation to precipitate proteins. The absorbance of the supernatant was then measured at 440 nm.

#### 2.5.5. Monoamine Oxidase Activity

The enzyme assay was conducted by incubating the homogenate with 0.5 mM kynuramine (Sigma-Aldrich, St. Louis, MO, USA) 50 mM phosphate buffer (pH 7.4) at 37 °C for 30 min, following Reis and Binda [45]. The reaction was terminated with the addition of 1 M NaOH, and the formation of 4-hydroxyquinoline was quantified spectrophotometrically at 316 nm under alkaline conditions. Parallel control assays containing MAO inhibitors were included to confirm reaction specificity. Enzymatic activity was calculated as the amount of product formed (nmol min^−1^ mg^−1^ protein).

#### 2.5.6. Neuropeptide F

Neuropeptides were extracted from pooled insect samples collected from both control and treated groups. The samples were homogenized and treated with acidified acetone (40:6:1, acetone: water: HCl, *v*/*v*/*v*), followed by centrifugation to obtain the supernatant. The resulting extract was evaporated to dryness and subsequently reconstituted in assay buffer. Quantitative analysis of neuropeptides was performed using an NPF ELISA kit (Catalog No. MBS2023813; My BioSource, Inc., San Diego, CA, USA) according to the manufacturer’s instructions.

### 2.6. Gas Chromatography–Mass Spectrometry (GC-MS) Analysis

The phytochemical composition of *A. herba alba* was evaluated for both the crude extract and tablet-like formulation to determine the retention of bioactive constituents. Prior to GC–MS injection, the samples were prepared as follows: 100 mg of the crude extract or 200 mg of the pulverized tablet formulation were dissolved in 10 mL of GC-grade n-ethanol (Sigma-Aldrich, St. Louis, MO, USA). The mixture was subjected to ultrasonic extraction using an ultrasonic cleaner (Elma Schmidbauer GmbH, Singen, Germany) for 15 min at room temperature to facilitate efficient extraction of phytochemicals. The resulting solution was filtered through a 0.22 µm PTFE syringe filter (Membrane Solutions, Auburn, WA, USA) to remove excipient residues (MCC and starch). Finally, 1 µL of the clear filtrate was injected into the GC–MS system using an AS1300 autosampler (Thermo Fisher Scientific, Waltham, MA, USA). Analyses were conducted using a Thermo Scientific TRACE 1310 GC–ISQ GC–MS system, equipped with a TG–5MS capillary column (30 m × 0.25 mm × 0.25 µm film thickness (Thermo Fisher Scientific, Waltham, MA, USA) under standardized operating conditions. The oven temperature program began at 50 °C, ramped at 5 °C min^−1^ to 230 °C (held for 2 min), and then increased at 30 °C min^−1^ to 290 °C, maintained for an additional 2 min. The injector and transfer line were set at 250 °C and 260 °C, respectively. Helium was used as the carrier gas at a constant flow rate of 1 mL min^−1^. A three-minute solvent delay was applied, and 1 µL of the diluted extract was injected automatically in split mode using an AS1300 autosampler. Ionization was achieved by electron impact (EI) at 70 eV, and spectra were collected in full-scan mode across an *m*/*z* range of 40–1000, with the ion source maintained at 200 °C. Compound identification was achieved by matching the mass spectra and retention indices with reference data from standard libraries, including the NIST/EPA/NIH Mass Spectral Library (NIST 2023 version) and the Wiley Registry of Mass Spectral Data.

### 2.7. Statistical Analysis

All data are presented as mean ± standard deviation (SD), and data distribution was assessed for normality prior to statistical analysis. Toxicity and repellency data were analyzed using two-way analysis of variance (ANOVA), whereas one-way ANOVA was applied for oxygen consumption and enzymatic activity assays. Significant differences among means were determined using Tukey’s post hoc test. Statistical significance was set at *p* < 0.05. Data organization and statistical analyses were performed using GraphPad Prism (version 8, GraphPad Software, San Diego, CA, USA), while Microsoft Excel (Microsoft Corp., Washington, DC, USA) was used for preliminary data handling. LC_50_ values were estimated using probit analysis based on dose–mortality data, following Finney’s method implemented in an Excel-based calculator (version 2021).

## 3. Results

### 3.1. Physicochemical Characterization of Tablet-like Formulations

The physicochemical evaluation of the formulated *A. herba-alba* tablet-like units showed consistent mechanical and physical stability across all formulations. Hardness values ranged from 5.29 ± 0.36 to 6.51 ± 0.25 N. Friability testing revealed low weight loss ranging from 0.73 ± 0.02% to 0.85 ± 0.03%, demonstrating good resistance to mechanical abrasion. Additionally, weight loss under desiccation conditions ranged from 1.53 ± 0.08% to 1.63 ± 0.05%, indicating minimal moisture-related mass change.

### 3.2. Comparative Insecticidal Activity of Crude Extract and Tablet-like Formulations

**Figure 1:**
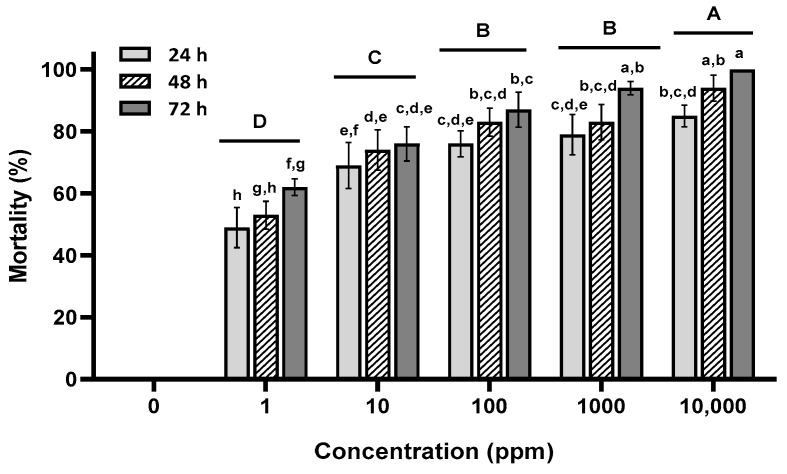
Mortality rates (mean % ± SD) of *Sitophilus oryzae* adults at 24, 48, and 72 h post exposure to various concentrations of *Artemisia herba-alba* crude extract. Bars marked with different letters indicate statistically significant differences among treatments according to Tukey’s test (*p* < 0.05).

**Figure 2:**
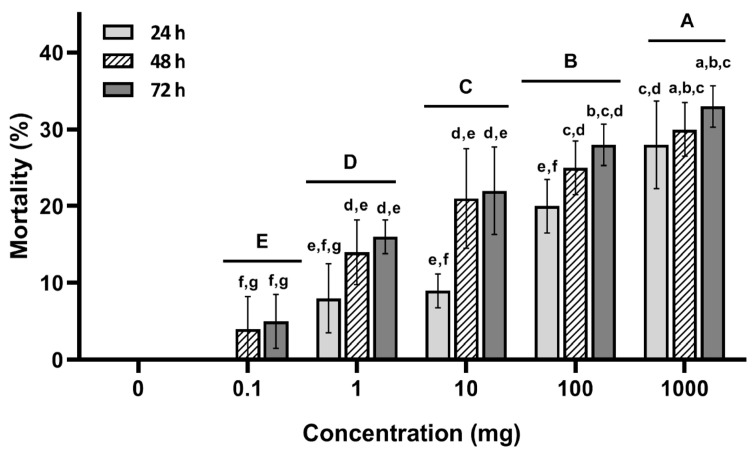
Mortality rates (mean % ± SD) of *Sitophilus oryzae* adults at 24, 48, and 72 h post-exposure to different extract-equivalent amounts of *Artemisia herba-alba* formulated as tablets. Bars marked with different letters indicate statistically significant differences among treatments according to Tukey’s test (*p* < 0.05).

The results showed that no mortality was observed in the control groups throughout the experimental period. The ethanolic extract of *A. herba-alba* exhibited a clear concentration- and time-dependent insecticidal effect against *S. oryzae*, with mortality significantly influenced by concentration (F = 128.7; df = 4; *p* < 0.0001) and exposure time (F = 36.99; df = 2; *p* < 0.0001). Mortality increased progressively from 62% at 1 ppm to 100% at 10,000 ppm after 72 h (Figure 1). A similar concentration (F = 106.1; df = 4; *p* < 0.0001) and time-dependence (F = 26.08; df = 2; *p* < 0.0001) trend was observed for the tablet-like formulation (Figure 2), although the overall mortality levels were markedly lower. At the lowest extract-equivalent level (0.1 mg), no mortality was recorded at 24 h, while mortality increased gradually to 33% at 1000 mg after 72 h. Overall, the crude extract exhibited higher insecticidal activity and a faster response compared to the tablet-like formulation (F = 6600.01; df = 1; *p* < 0.001). LC_50_ values (Table 1 and Table 2) further confirmed the substantial difference in potency between the two treatments across all exposure times, with the crude extract demonstrating markedly greater toxicity than the tablet-like formulation.

### 3.3. Repellency Assay

**Figure 3:**
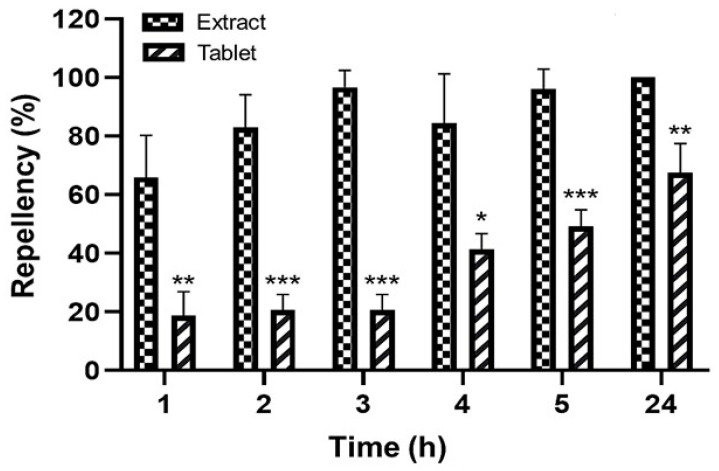
Repellent activity (% mean ± SD) of *Artemisia herba-alba* ethanolic crude extract and tablet-like formulation against *Sitophilus oryzae* adults at a sublethal exposure level (equivalent to 1/10 of the 24 h LC_50_) over different time intervals. Statistical significance between formulations at each exposure time is indicated by asterisks above the bars: * *p* < 0.05, ** *p* < 0.01, *** *p* < 0.001.

As illustrated in Figure 3, the crude extract of *A. herba-alba* demonstrated a highly significant repellent effect against *S. oryzae* compared to the tablet-like formulation (F = 605.9; df = 1; *p* < 0.0001), with repellency increasing progressively over time (F = 14.48; df = 5; *p* = 0.0019). For the crude extract, repellency reached 65.92% after 1 h and 82.96% after 2 h, and peaked at 96.66% after 3 h. A slight decline was observed at 4 h (84.44%), followed by a recovery to 96.07% at 5 h, and ultimately reaching complete repellency (100%) after 24 h. In contrast, the tablet-like formulation displayed minimal initial repellency, reaching only 18.77% at 1 h and increasing gradually over time, reaching a maximum 67.44% after 24 h. These results suggest that the crude extract induced a rapid and potent repellent effect, whereas embedding the equivalent concentration into a tablet-like form led to a slower and less pronounced repellent response.

### 3.4. Effect of Treatments on Oxygen Consumption Rate

**Figure 4:**
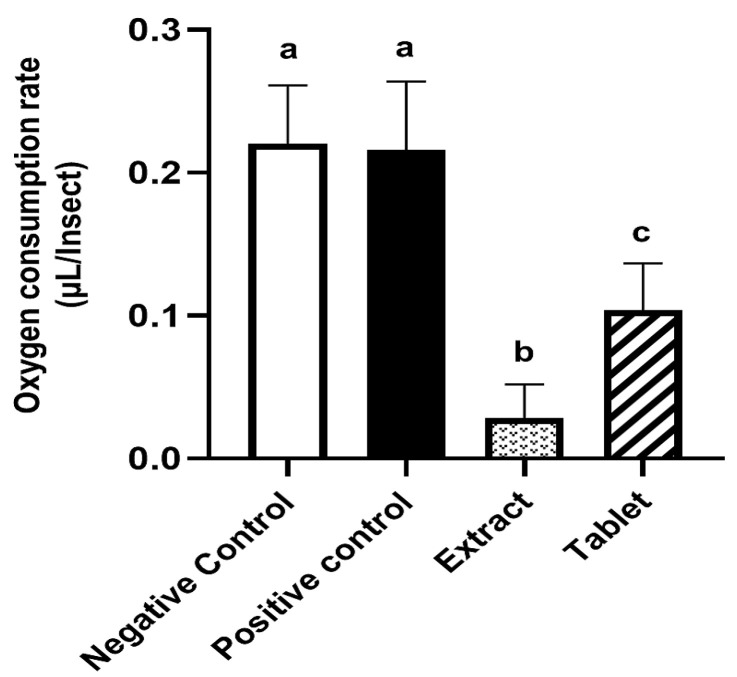
Oxygen consumption rate (mean µL ± SD) of *Sitophilus oryzae* adults following treatment with *Artemisia herba-alba* crude extract and tablet-like formulations at a sublethal exposure level (equivalent to 1/10 of the calculated 24 h LC_50_). Columns labeled with different letters indicate significant differences at *p* < 0.05.

The results demonstrated that both formulations of *A. herba-alba* significantly reduced oxygen consumption in *S. oryzae* compared to the control group (F = 31.12; df = 3; *p* < 0.0001) (Figure 4). However, the crude extract had a stronger inhibitory effect on respiration than the equivalent amount formulated in a solid tablet-like form (*p* = 0.0260).

### 3.5. Effect of Treatments on Biochemical Parameters 

**Figure 5:**
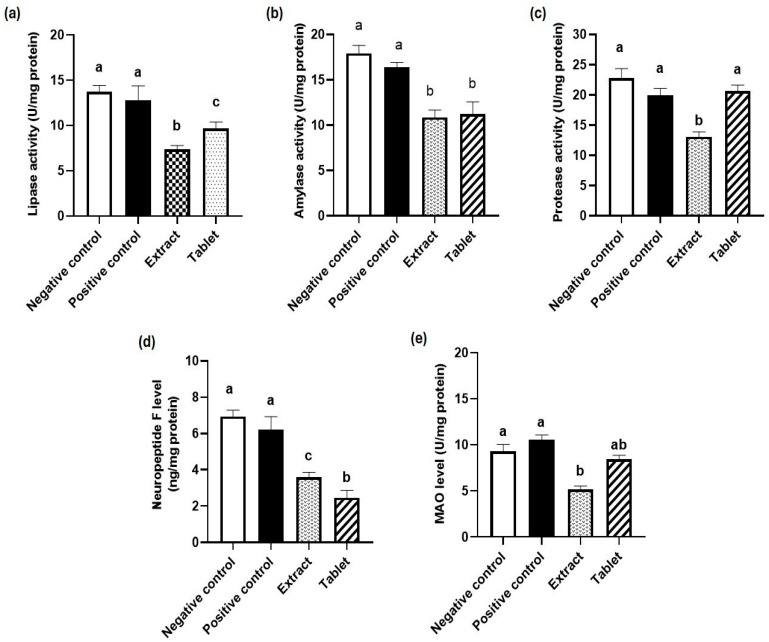
Effects of a sublethal exposure level (equivalent to 1/10 of the calculated 24 h LC_50_) of *Artemisia herba-alba* crude extract and tablet-like formulations on biochemical parameters of *Sitophilus oryzae*. (**a**) lipase activity, (**b**) α-amylase activity, (**c**) protease activity, (**d**) neuropeptide F (NPF) level, and (**e**) monoamine oxidase (MAO) activity. Data are presented as mean ± SD. Columns not sharing the same letter within the physiological parameter are significantly different at *p* < 0.05.

As illustrated in Figure 5, both the crude extract and tablet-like formulations of *A. herba-alba* significantly decreased lipase (Figure 5a; F = 28.43, df = 3, *p* < 0.0001) and α-amylase (Figure 5b; F = 42.28, df = 3, *p* < 0.0001) activities in *S. oryzae* compared to the control. However, protease activity (Figure 5c) was only significantly reduced in insects treated with the crude extract (F = 37.96, df = 3, *p* < 0.0001). NPF levels (Figure 5d) were significantly decreased by both formulations relative to the control (F = 60.46, df = 3, *p* < 0.0001). In contrast, MAO activity was significantly inhibited only in insects exposed to the crude extract (*p* = 0.0003) (Figure 5e). Overall, the crude extract had a broader inhibitory influence, affecting both digestive enzymes and signaling-related parameters, while the equivalent amount of extract in the tablet-like formulation showed a more selective effect.

### 3.6. GC–MS Analysis

The GC–MS analysis of *A. herba-alba* revealed a chemically diverse composition in both the crude ethanolic extract and the tablet-like formulation (Table 3 and Table 4), respectively. The crude extract contained 34 identifiable compounds, with major constituents including 1H-indole-3-ethanamine, N, N-dimethyl (51.70%), dopamine N, N-dimethyl derivative (23.41%), and other N-methylphenethylamine derivatives. Additionally, the extract contained a variety of fatty acid esters, terpenoids, phenolic compounds, and minor alkaloids, indicating a complex phytochemical profile. In comparison, the tablet-like formulation contained 39 compounds, with R, R(-)-Pseudoephedrine TMS derivative (64.37%) and Paredrine TMS derivative (8.84%) as the predominant constituents. Other compounds detected included fatty acid methyl esters, alcohol, and minor phenethylamine derivatives.

A comparison between the crude extract and the tablet-like formulation revealed several shared compounds (Table 5), such as 1H-indole-3-ethanamine, N,N-dimethyl, Benzene methanol, α-[1-(methylamino)ethyl]-, 3-Hydroxy-N-methylphenethylamine, and selected fatty acid methyl esters including 13,16-Octadecadiynoic acid, 9-Octadecenoic acid (Z)-, and 6-Octadecenoic acid (Z)-. These compounds were present in both matrices, but their relative abundances were lower in the tablet formulation.

## 4. Discussion

The development of botanical insecticides has shifted increasingly from searching for bioactive plant species towards optimizing formulation systems. Therefore, evaluating the same botanical material in different formulation forms is crucial not only for understanding formulation-dependent changes in efficacy but also for identifying the most appropriate delivery strategy for specific pest management objectives. In this context, the present study provides a comprehensive comparison between crude and tablet-like formulations of *A. herba-alba*, integrating biological, physiological, and phytochemical assessments against *S. oryzae*.

The physicochemical characterization of the tablet-like formulation demonstrated satisfactory mechanical integrity and structural stability of the plant-based matrix. The observed hardness and friability values are consistent with previously reported ranges for similar herbal solid systems [27], indicating adequate mechanical cohesion and resistance to structural breakdown under stress. In addition, the low weight loss under desiccation conditions reflects minimal residual moisture and stable physical properties of the formulations, in agreement with reported requirements for the stability of herbal solid preparations [46]. Overall, these findings support the formation of a stable plant-derived matrix suitable for handling and subsequent bioassays.

The results demonstrated that the crude extract achieved complete insect mortality (100%), whereas the tablet formulation induced a delayed and lower mortality (33%). These findings align with those reported by Jaber et al. [47], who showed that the ethanolic extract of *A. herba-alba* caused high mortality rates, reaching up to 92% against *T. castaneum*. In contrast, Kathirvelu et al. [26], who evaluated tablet formulations of several botanicals including *Artemisia vulgaris*, against the Adzuki bean weevil (*Callosobruchus chinensis* L., Coleoptera: Chrysomelidae) and the lesser grain borer (*Rhyzopertha dominica* F., Coleoptera: Bostrichidae), reported mortality rates not exceeding 50%.

The pronounced difference between the two formulations may be attributed to variations in the mode of exposure and release kinetics of the bio-active constituents. In the crude extract, the rapid release of volatile and semi-volatile compounds leads to a high concentration of active molecules within the confined space, suggesting that inhalation toxicity is the dominant mechanism, with a secondary contribution from contact toxicity through surface deposition. In contrast, the tablet-like formulation functions as a controlled-release system, where compaction of plant material reduces the rate of volatilization. This likely limits the concentration of airborne toxicants, thereby decreasing fumigant toxicity and shifting the mode of action toward slower contact-mediated effects [22,48].

Regarding repellency, a clear difference was observed between the crude extract and the tablet-like formulation, where the crude extract induced complete repellency (100%) within 24 h, whereas the tablet-like formulation exhibited a lower short-term response (67.44%). This indicates that the formulation process may influence the immediate behavioral effect against *S. oryzae*. This difference can be explained by variations in the chemical profile, relative abundance, and volatility of bioactive constituents as demonstrated by GC–MS analysis. The crude extract was characterized by a diverse mixture of volatile and semi-volatile constituents, including nitrogen-containing aromatic derivatives and phenethylamine-related compounds, alongside volatile monoterpene constituents such as limonene. These compounds are known for their high volatility and rapid diffusion, which facilitate efficient interaction with insect olfactory receptors and contribute to strong behavioral repellency responses. In contrast, the tablet-like formulation displayed a compositional shift toward less volatile and more chemically stable compounds, including fatty acid methyl esters and long-chain aliphatic derivatives. This shift is likely associated with formulation-related effects such as matrix entrapment and altered release dynamics, which may reduce the immediate vapor-phase availability of highly volatile constituents [22,49].

Mechanistically, plant-derived insecticides are known to exert their effects through multiple pathways, including neurotoxicity, disruption of respiratory processes, and interference with growth regulation [50]. In the present study, both formulations significantly reduced oxygen consumption, indicating an impairment of insect respiratory metabolism. This reduction suggests a potential disruption of mitochondrial function, possibly through uncoupling of oxidative phosphorylation and/or interference with spiracular regulation, ultimately leading to decreased energy production and impaired physiological performance. Similar inhibitory effects on insect respiration have been previously reported for *Artemisia*-based products and other botanical formulations [51,52,53].

The biochemical assays further demonstrated that both the crude extract and the tablet formulation significantly reduced amylase, lipase, and NPF levels. However, inhibition of protease and MAO activity was observed only in insects treated with the crude extract. The shared reduction in amylase, lipase, and NPF levels in both treatments may be associated with common bioactive constituents, including 1H-indole-3-ethanamine, N,N-dimethyl, and 3-hydroxy-N-methylphenethylamine. These compounds belong to the indole and phenethylamine classes and are structurally related to biogenic amines, which are reported to modulate insect neurophysiology and feeding-related signaling pathways [54,55]. In addition, both formulations contained fatty acid methyl esters, including 9-octadecenoic acid (Z)-, methyl ester and 6-octadecenoic acid methyl ester, which have been reported to interfere with membrane integrity and physiological processes. Such effects may contribute to the observed reduction in amylase and lipase activities [56,57].

In contrast, the selective inhibition of protease and MAO activity observed only in the crude extract may be attributed to the presence of additional bioactive constituents, particularly phenolic compounds such as phenol, 4-(2-aminopropyl) and amine-related compounds including hordenine and dopamine-like derivatives. Phenolic compounds are known to interact with protein structures, leading to inhibition of proteolytic enzymes such as proteases [58], while amine-containing compounds may interfere with MAO activity due to their structural similarity to endogenous substrates [59]. Collectively, these findings indicate that the biological performance of botanical insecticides is strongly modulated by formulation design, which governs compound availability and exposure dynamics, and therefore represents a key determinant of their overall efficacy beyond chemical composition alone.

## 5. Conclusions

While numerous previous studies have promoted tablet formulations as superior delivery systems for botanical insecticides due to their controlled release and enhanced stability, the present study demonstrates that this assumption cannot be generalized to all plant species and formulations. In the case of *A. herba-alba*, the crude extract exhibited greater biological activity against *S. oryzae* than the tablet-like formulation, particularly for the most critical parameter, insect mortality, where faster and higher toxic effects were observed. Nevertheless, the tablet formulation showed comparable performance in several other parameters, including repellency, respiration rate, and certain physiological responses. These findings indicate that formulation selection should not be based solely on the perceived superiority of a delivery system, but rather on the specific pest management objective. Accordingly, the crude extract may be the preferred option when rapid control is required under high infestation levels, whereas the tablet formulation may be more suitable for preventive applications, repellency, and the maintenance of grain quality during storage.

Despite these promising findings, this study has some limitations. Experiments were conducted under controlled laboratory conditions, which may not fully reflect the complexity of real storage environments. In addition, the long-term stability and persistence of both formulations were not evaluated, and the proposed mechanisms underlying their biological activity were not confirmed at the molecular level. Therefore, further investigations under field or semi-field storage conditions, over extended exposure periods, and incorporating molecular analyses are necessary to validate the practical applicability and mode of action of both formulations in stored-grain protection systems.

## Figures and Tables

**Table 1:** Median lethal concentrations (LC_50_) required to achieve 50% mortality of adult *Sitophilus oryzae* at 24, 48, and 72 h post-exposure to various concentrations of *Artemisia herba-alba* crude extract.

Time (h)	LC_50_ (ppm)	95% Confidence Limits	Slope	
24	0.33684	0.00945	12.00297	0.243
48	0.28000	0.01629	4.81353	0.324
72	0.19273	0.01639	2.26622	0.417

**Table 2:** Median lethal concentrations (LC_50_) of extract-equivalent amounts of *Artemisia herba-alba* embedded in tablet-like formulations against adult *Sitophilus oryzae* at 24, 48, and 72 h post-exposure.

Time (h)	LC_50_ (mg of Extract/Tablet)	95% Confidence Limits	Slope	
24	107,427.84060	3161.03258	3,650,940.203	0.294
48	26,825.87360	923.29903	779,408.917	0.278
72	15,100.85107	542.08089	420,667.298	0.276

**Table 3:** Chemical constituents of *Artemisia herba alba* ethanolic extract.

No.	Compound Name	Retention Time	Area %	Molecular Formula	Retention Indices
1	2-Methyl-2-propanamine	7.45	4.27	C_4_H_11_N	501
2	2-Methoxy-N,N-dimethyleth anamine	7.45	4.27	C_5_H_13_NO	643
3	1H-indole-3-ethanamine, N,N-dimethyl	16.99	51.70	C_10_H_15_NO	1715
4	Dopamine, N,N-dimethyl-, dimethyl ether	16.68	23.41	C_12_H_19_NO_2_	1615
5	Hordenine	16.99	51.70	C_10_H_15_NO	1495
6	Dopamine, N,N-dimethyl-, dimethyl ether	16.99	51.70	C_12_H_19_NO_2_	1615
7	Benzenemethanol, à-[1-(methylamino)ethyl]-, [R-(R*,S*)]-	16.99	51.70	C_10_H_15_NO_2_	1269
8	2-(Methylamino)-1-phenyl-1 -propanol	16.99	51.70	C_10_H_15_NO	1389
9	3-Hydroxy-N-methylphenethylamine	18.00	6.48	C_9_H_13_NO	1412
10	Phenol, 4-(2-aminopropyl)-, (.+−.)	17.83	1.18	C_9_H_13_NO	1429
11	2-[Benzyloxyimino]methyl-3-methyl-1-(1-propanesulfonate)imidazolium inner salt	18.00	6.48	C_15_H_19_N_3_O_4_S	1634
12	Paradrine	18.00	6.48	C_9_H_13_NO	1404
13	Phenol, 4-(2-aminopropyl)-, (.+−.)-	18.00	6.48	C_9_H_13_NO	1429
14	Phenol, 4-(2-aminoethyl)-	18.00	6.48	C_8_H_11_NO	1430
15	1,4-Benzenedimethanol	23.20	0.76	C_8_H_10_O_2_	1404
16	1,3-Cyclooctadiene, 5-bromo-	23.20	0.76	C_8_H_11_Br	1182
17	2,7-Dioxa-tricyclo[4.4.0.0(3,8)]deca4,9-diene	23.26	0.20	C_8_H_8_O_2_	841
18	3-Hydroxy-N-methylphenethylamine	23.57	0.14	C_9_H_13_NO	1412
19	2-[Benzyloxyimino]methyl-3-methyl-1-(1-propanesulfonate)imidazolium inner salt	23.57	0.14	C_15_H_19_N_3_O_4_S	1319
20	Phenol, 4-(2-aminoethyl)-	23.57	0.14	C_8_H_11_NO	1430
21	1,4-Benzenedimethanol	23.57	0.14	C_8_H_10_O_2_	1392
22	3-Hydroxy-N-methylphenethylamine	23.61	0.20	C_9_H_13_NO	1412
23	Phenol, 4-(2-aminopropyl)-, (.+−.)-	23.61	0.20	C_9_H_13_NO	1429
24	4-(1H-Imidazol-1-ylmethyl)phenol	23.61	0.20	C_10_H_10_N_2_O	1609
25	Phosphinic acid, di(phenoxymethyl)	23.86	0.99	C_14_H1_5_O_4_P	NA
26	13,16-Octadecadiynoic acid, methyl ester	26.41	0.89	C_19_H_30_O_2_	2112
27	12-Tridecynoic acid, methyl ester	26.41	0.89	C_14_H_24_O_2_	1578
28	9-Octadecenoic acid (Z)-, methyl ester	29.62	0.45	C_19_H_36_O_2_	2113
29	(9E,12E)-9,12-Octadecadieno YL chloride	29.62	0.45	C_18_H_31_ClO	2139
30	6-Octadecenoic acid, methyl ester, (Z)-	29.62	0.45	C_19_H_36_O_2_	2081
31	R-Limonene	30.82	1.50	C_10_H_16_O_3_	1018
32	Tricyclo[8.6.0.0(2,9)]Hexadec A-3,16-Dion, Trans-2,9-ransoid-9,10-Cis-1,10	30.82	1.50	C_16_H_24_O_2_	1687
33	Cyclooctenone, Dimer	30.82	1.50	C_16_H_24_O_2_	904
34	Aspidospermidin-17-OL, 1-acetyl-16-methoxy	30.82	1.50	C_22_H_30_N_2_O_3_	2924

NA means that the retention index value for this compound is not available.

**Table 4:** Chemical constituents of *Artemisia herba alba* tablet-like formulation.

No	Compound Name	Retention Time	Area %	Molecular Formula	Retention Indices
1	Benzenemethanol,4-hydroxy-à-[1-(methylamino)ethyl]-, (R*,S*)-	15.56	1.60	C_10_H_15_NO_2_	1357
2	1H-Indole-3-ethanamine,N,N-dimethyl-	15.56	1.60	C_10_H_15_NO	1715
3	1-(1,3-Benzodioxol-4-yl)-N-methylpropan-2-amine	15.56	1.60	C_11_H_15_NO_2_	1570
4	Benzenemethanol,à-[1-(methylamino)ethyl]-,[S-(R*,R*)]-	15.63	1.52	C_10_H_15_N	1743
5	Paredrine, TMS derivative	15.93	8.84	C_12_H_21_NOSi	1469
6	R, R(-)-Pseudoephedrine, trimethylsilyl ether	16.32	64.37	C_13_H_23_NOSi	1446
7	9-Octadecenoic acid, (2-phenyl-1,3-dioxolan-4-yl)methyl ester, CIS-	22.73	2.58	C_28_H_44_O_4_	2019
8	9,10-Secocholesta-5,7,10(19)-triene-1,3-diol, 25-[(trimethylsilyl)oxy]-, (3á,5Z,7E)-	22.73	2.58	C_30_H_52_O_3_Si	2806
9	Limonen-6-ol, pivalate	22.73	2.58	C_15_H_24_O_2_	1560
10	2,3-Di(2,2-dimethylethyl) thiophene-1,1-dioxide	22.96	1.28	C_12_H_20_O_2_S	2076
11	1,3-Dioxolane, 2-phenyl-	22.96	1.28	C_9_H_10_O_2_	1215
12	(2-methyl-3-nitrophenyl)methanol	22.96	1.28	C_8_H_9_NO_3_	2341
13	3-Bromo-5-methyl-1-adamantanecarboxylic acid	22.96	1.28	C_12_H_17_BrO_2_	1654
14	4a-Dichloromethyl-4,4a,5,6,7,8-hexahydro-3H-naphthalen-2-one	23.04	0.87	C_11_H_14_Cl_2_O	2226
15	3-Hydroxy-N-methylphenethylamine	24.45	2.53	C_9_H_13_NO	1412
16	Benzenemethanol,4-hydroxy-à-(methylamino)methyl]-	24.45	2.53	C_9_H_13_NO_2_	1357
17	Methyl N-(N-benzyloxycarbonyl-beta-l-aspartyl)-beta-d-glucosaminide	24.45	2.53	C_19_H_26_N_2_O_10_	3815
18	2H-Pyran, tetrahydro-2-(2,5-undecadiynyloxy)-	24.45	2.53	C_16_H_24_O_2_	2254
19	Cyclopropanebutanoic acid, 2-[[2-[[2-[(2-pentylcyclopropyl)methyl]cyclopropyl]methyl]cyclopropyl] methyl]-, methyl ester	24.68	2.30	C_25_H_42_O_2_	2528
20	14-Pentadecynoic acid,methyl ester	24.68	2.30	C_16_H_28_O_2_	1859
21	13,16-Octadecadiynoic acid, methyl ester	24.68	2.30	C_19_H_30_O_2_	2112
22	9,12,15-Octadecatrienoic acid, 2,3 bis(acetyloxy)propyl ester, (Z,Z,Z)	25.64	1.17	C_25_H_40_O_6_	2058
23	N-[4-(4-Chlorophenyl)isothiazol-5-yl)-1-methylpiperidin-2-imine	25.64	1.17	C_15_H_16_ClN_3_S	2580
24	Dihydroneotigogenin 26-tosylate	25.64	1.17	C_34_H_52_O_5_S	2906
25	9-octadecenoic acid (Z)-	27.83	1.76	C_18_H_34_O_2_	2113
26	Cyclopropanedodecanoic acid,2-octyl-, methyl ester	27.83	1.76	C_24_H_46_O_2_	2538
27	7-Hexadecenoic acid, methyl ester,(Z)-	27.83	1.76	C_17_H_32_O_2_	1888
28	9-Octadecenoic acid (Z)-, methyl ester	27.83	1.76	C_19_H_36_O_2_	2082
29	6-Octadecenoic acid, methyl ester, (Z)	27.97	2.03	C_19_H_36_O_2_	2081
30	(9E,12E)-9,12-Octadecadienoyl chloride	27.97	2.03	C_18_H_31_ClO	2139
31	9,12-Octadecadienal, dimethyl acetal	29.94	1.72	C_20_H_38_O_2_	2422
32	2-Aminoethanethiol hydrogen sulfate (ester)	29.94	1.72	C_2_H_7_NO_3_S_2_	NA
33	Isochiapin B	31.62	0.85	C_19_H_22_O_6_	2030
34	2-Acetyl-3-(2-cinnamido)ethyl-7-methoxyindole	33.23	1.09	C_22_H_22_N_2_O_3_	2624
35	1,3,5-Triazine-2,4-diamine, 6-chloro-n-ethyl-	33.23	1.09	C_5_H_8_ClN_5_	1285
36	Dotriacontane	33.23	1.09	C_32_H_66_	3200
37	4H-1-Benzopyran-4-one, 2-(3,4 dimethoxyphenyl)-3,5-dihydroxy-7-methoxy	34.80	1.57	C_18_H_16_O_7_	3269
38	1-Heptatriacotanol	39.24	2.21	C_37_H_76_O	3942
39	2-Nonadecanone 2,4-dinitrophenylhydrazine	39.77	1.70	C_25_H_42_N_4_O_4_	3765

NA means that the retention index value for this compound is not available.

**Table 5:** Bioactive constituents shared in crude extract and tablet-like formulations of *Artemisia herba alba*.

Compound Name	RT (Crude)	RT (Tablet)	Area % (Crude)	Area % (Tablet)	Molecular Formula
1H-indole-3-ethanamine, N,N-dimethyl	16.99	15.56	51.70	1.60	C_10_H_15_NO
Benzenemethanol, à-[1-(methylamino)ethyl]-, [R-(R*,S*)]-	16.99	15.56	51.70	1.60	C_10_H_15_NO_2_
3-Hydroxy-N-methylphenethylamine	18.00	24.45	6.48	2.53	C_9_H_13_NO
13,16-Octadecadiynoic acid, methyl ester	26.41	24.68	0.89	2.30	C_19_H_30_O_2_
9-Octadecenoic acid (Z)-, methyl ester	29.62	27.83	0.45	1.76	C_19_H_36_O_2_
6-Octadecenoic acid, methyl ester, (Z)-	29.62	27.97	0.45	2.03	C_19_H_36_O_2_

## Data Availability

The data that supports the findings of this study are available from the corresponding author upon request.
